# One-lung flooding reduces the ipsilateral diaphragm motion during mechanical ventilation

**DOI:** 10.1186/s40001-016-0205-1

**Published:** 2016-03-08

**Authors:** Thomas Günther Lesser, Harald Schubert, Daniel Güllmar, Jürgen R. Reichenbach, Frank Wolfram

**Affiliations:** Department of Thoracic and Vascular Surgery, SRH Wald-Klinikum Gera, Teaching Hospital of Friedrich-Schiller University of Jena, Strasse des Friedens 122, 07548 Gera, Germany; Institute of Animal Experimentation and Animal Welfare, Jena University Hospital, Friedrich-Schiller University, Jena, Germany; Medical Physics Group, Institute of Diagnostic and Interventional Radiology, Jena University Hospital, Friedrich-Schiller University, Jena, Germany

**Keywords:** Liver or lung tumours, HIFU, Lung flooding, Diaphragm motion, Magnetic resonance imaging

## Abstract

**Background:**

Diaphragm motion during spontaneous or mechanical respiration hinders image-guided percutaneous interventions of tumours in lung and upper abdomen. Motion-tracking methods can be applied but increase procedure complexity and procedure time. One-lung flooding (OLF) generates a suitable acoustic pathway to lung tumours and likely suppress diaphragm motion. The aim of this study was to quantify the effect of OLF on ipsilateral diaphragm motion during contralateral one-lung ventilation.

**Methods:**

To measure the diaphragm motion, M-mode ultrasonography of the right hemidiaphragm was performed during spontaneous breathing and mechanical ventilation, as well as after right-side lung flooding, in three pigs. Diaphragm motion was analysed using magnetic resonance images during left-side lung flooding and mechanical ventilation, in four pigs.

**Results:**

Double-lung ventilation increased the diaphragm movement in comparison with spontaneous breathing (17.8 ± 4.4 vs. 12.2 ± 3.4 mm, *p* = 0.014). Diaphragm movement on the flooded side during contralateral one-lung ventilation was significantly reduced compared to that during double-lung ventilation (3.9 ± 1.0 vs. 17.8 ± 4.4 mm, *p* = 0.041). By analysing the magnetic resonance images, the hemidiaphragm on the flooded side showed an average displacement of 4.2 mm, a maximum displacement of 15 mm close to the ventilated lung and no displacement at the lateral side.

**Conclusion:**

OLF leads to a drastic reduction of diaphragm motion on the ipsilateral side which implies that targeting and motion compensation algorithms for interventions like high-intensity focused ultrasound ablation of intrapulmonary and hepatic lesions might not be required.

## Background

The respiratory motion of the upper abdominal organs is mainly caused by diaphragm motion. Organ motion requires gating of imaging techniques and hinders the precise targeting of interventions in lung and upper abdomen [1, 2]. Extracorporeal high-intensity focused ultrasound (HIFU) is a relatively new non-invasive method for ablation of tumours in deeply located organs in the upper abdomen [3, 4]. Currently the accurate respiratory motion compensation for moving organs is a great challenge. In magnetic resonance-guided HIFU (MRgHIFU) the organ motion requires active target tracing for steering the HIFU beam as well as multibaseline reconstruction methods for proton resonance frequency shift (PRFS) thermometry [5, 6]. Respiratory-gated MRgHIFU and MR thermometry using a pencil beam navigator on the diaphragm lead to very lengthy treatment times [7]. Real-time methods for motion-compensated MR thermometry and MRgHIFU show promising results, but are expensive and requiring additional computation and specialist equipment [6, 8]. Several motion compensation and target tracing techniques are based on 2D data, whereas local motion occurs in three dimensions.

Beside all of the available target tracking methods, a reduction of diaphragm motion and thereby avoidance of target motion is preferred. Special ventilation techniques, such as one-lung ventilation, high-frequency jet ventilation (HFJV) and high-frequency oscillatory ventilation (HFOV) can reduce movement of the diaphragm and thus avoid gating or motion compensation for MR imaging [9, 10].

One-lung flooding (OLF) with saline is a method to replace air with water content in lung parenchyma. Previous work has shown that OLF enables efficient lung sonography and tumour imaging in an in vivo porcine model [11]. Furthermore, the flooded lung represents an ideal acoustic path for HIFU insonation into lung tumours [12, 13]. The flooded lung is not ventilated and its mechanical properties are likely stiffer compared to ventilated condition. Therefore, it can be assumed that the diaphragmatic motion on the flooded side should be reduced, which will be beneficial for interventions that require OLF for an acoustic access to lung or upper abdomen.

Because OLF requires mechanical ventilation of the contralateral lung, the extent of the motion suppression of the hemidiaphragm on the flooded side should be investigated. The aim of this study was to quantify the effect of OLF on ipsilateral diaphragm motion during contralateral one-lung ventilation using an in vivo large animal model.

## Methods

### Anaesthesia and preparation

Animal experiments were performed on seven female pigs (Deutsches Landschwein breed; weight: 28–32 kg; average: 30 kg) with permission from the Veterinary Department of the Thuringian State Authority for Food Protection and Fair Trading in compliance with the National Animal Protection Act (TLLV). The animals were premedicated with ketamine (1200 mg i.m.) and midazolam (10 mg i.m.), and general anaesthesia was induced with sufentanil (1 µg kg^−1^) and propofol (3 mg kg^−1^) via a peripheral vein. Anaesthesia was maintained with a continuous infusion of propofol (10 mg kg^−1^ h^−1^) and sufentanil (0.02 μg kg^−1^ min^−1^). After the onset of anaesthesia, the pigs were intubated with a single-lumen endotracheal tube (Magill tube, inner diameter = 8.5 mm, Mallinckrodt™, Covidien, Neustadt, Germany). Following the first ultrasound examination during spontaneous breathing, the animals were relaxed (pancuronium bromide, 2.5 μg kg^−1^ min^−1^) and mechanically ventilated. Mechanical ventilation was performed with an ICU respirator (Servo 900, Siemens AG, Munich, Germany) on a volume-controlled setting (FIO_2_ = 0.4; tidal volume (TV) 10 ml kg^−1^ body weight with the respiratory rate adjusted to maintain an end tidal CO_2_ of 35–45 mmHg; I:E ratio 1:1; PEEP 5 cm H_2_O).

A left-sided Robertshaw double-lumen tube with an extra-long bronchial lane (size 39 Ch; special product by Mallinckrodt Medical, Dublin, Ireland) was inserted after tracheotomy. The correct position of the tube was checked by fibrebronchoscopy (BF 3C30; Olympus, Tokyo, Japan). The right internal jugular vein was dissected, and a three-lumen central venous catheter (certofix; B. Braun, Melsungen, Germany) was inserted. The right carotid artery was cannulated (Arterial Leader Cath 2.7 Fr; Vygon, Ecouen, France) to allow blood gas analysis and invasive measurement of blood pressure. The electrocardiogram, arterial blood pressure, capillary oxygen saturation and expiratory CO_2_ concentration were measured and recorded continuously (Datex AS/3 Compact Multiparameter Patient Monitor; Datex-Ohmeda Corp., Helsinki, Finland). Arterial blood gases were analysed every 30 min (ABL System 625; Radiometer Medical, Copenhagen, Denmark).

During MR examination vital parameters were monitored using pulse oximeter, respiration belt and MRI-compatible ECG sensors.

### Lung flooding

Thirty minutes after ventilation with FIO_2_ = 1.0, the right endobronchial tube leg was disconnected from the respirator. The infusion system was immediately connected to the right tube leg and the lung was slowly filled with 465 ml of degassed and tempered (35 °C) isotonic saline solution. This volume represents the functional residual capacity (FRC) and the TV of a porcine lung wing [14]. A single filling was performed passively using the gravity of the liquid flowing from an infusion bottle suspended 50 cm above heart level. During one-lung ventilation, the respirator settings remained unchanged. For MR examination the left lung was flooded in the same way.

### Measurement of diaphragm movement

#### Sonography

**Fig. 1:**
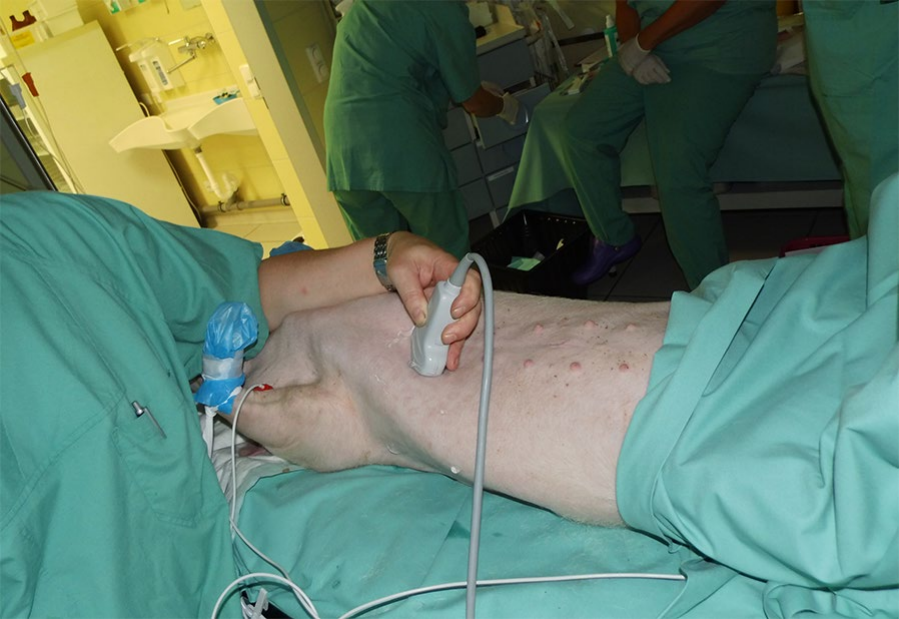
Subcostal approach for the measurement of movement of the right hemidiaphragm by ultrasound (curved array C60e, 5–2 MHz; MikroMaxx; SonoSite, Inc.)

**Fig. 2:**
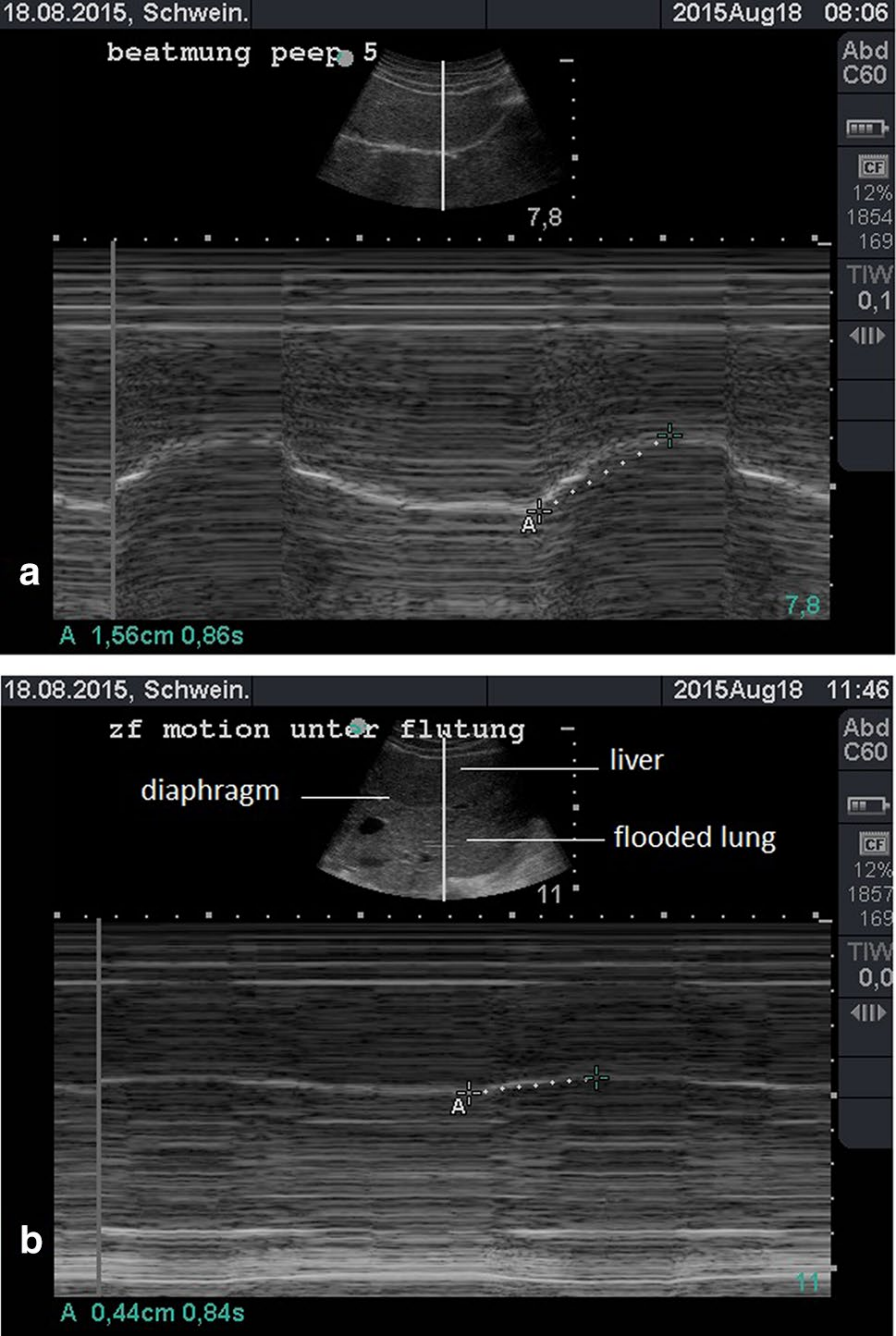
M-mode sonography for the measurement of diaphragm movement (end-expiration/end-inspiration difference) **a** during mechanical ventilation and **b** after lung flooding

Right hemidiaphragm movement was measured using M-mode ultrasonography during spontaneous breathing and mechanical ventilation of both lungs, as well as after right-side lung flooding in supine position, in three pigs. To assess the movement of the right hemidiaphragm, the diaphragm dome was visualised by ultrasound (curved array C60e, 5–2 MHz; MikroMaxx; SonoSite Inc., Bothell, WA, USA) using a subcostal approach (Fig. 1). In M-mode, a selected sound path with a beam angle ≥70° was used to follow changes in diaphragm dome position over time. The excursion amplitude between the end of expiration and the end of inspiration was measured three times. For that measurement, the greatest waveform was chosen (Fig. 2).

#### MR imaging

**Fig. 3:**
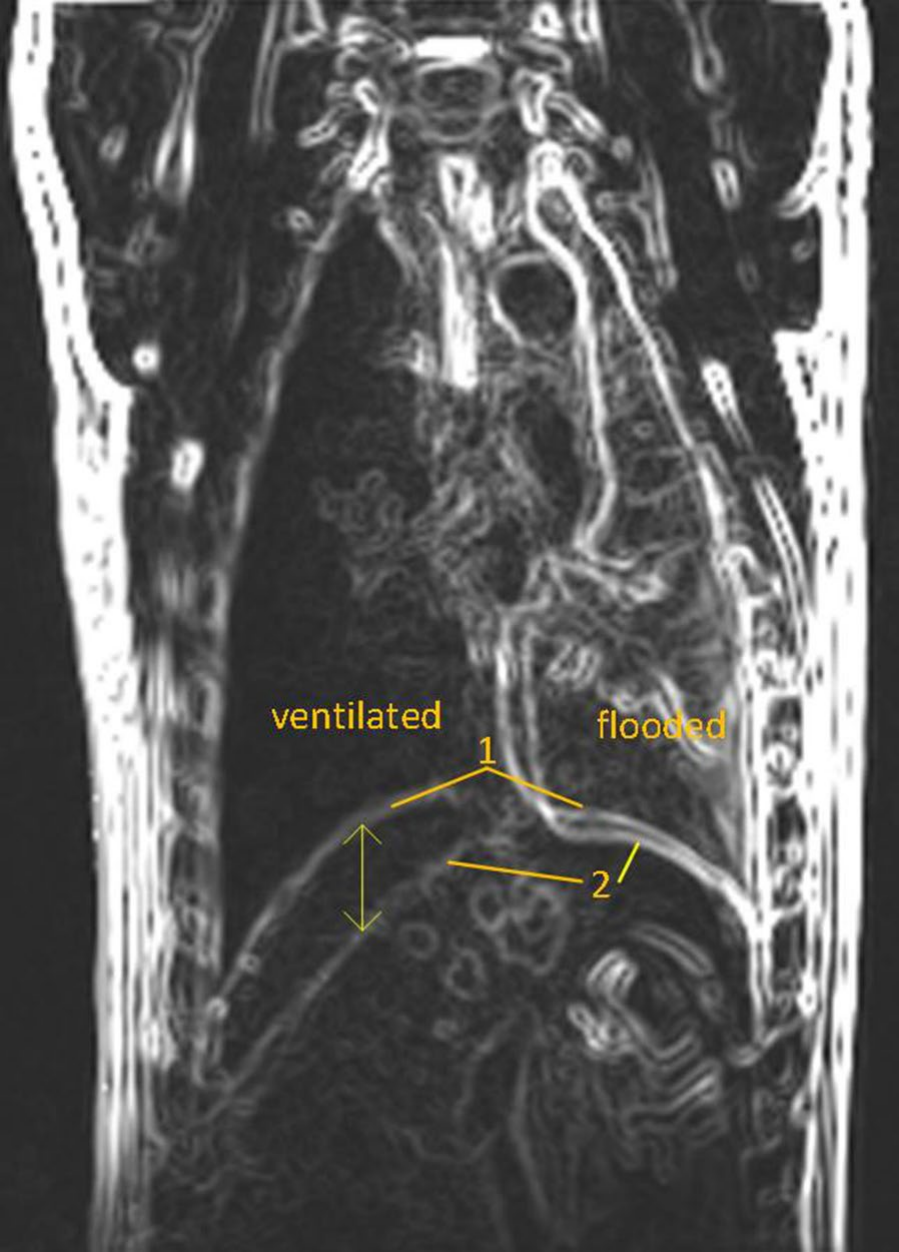
MR image merged from end-expiration and end-inspiration after edge detection; localisation of the flooded lung left and ventilated lung right with diaphragm position in end-expiration (*1*) and end-inspiration (*2*). *Arrow* indicates diaphragm displacement during breath cycle in the frontal plane

MR examinations were performed on four animals. After successful flooding of the left lung wing the animal was placed in 3 Tesla MRI clinical scanner (Prisma Fit, Siemens Medical Solutions, Erlangen, Germany) in lateral decubitus position, with flooded lung below. Mechanical ventilation of the right lung was maintained as described above. MR imaging was performed using spine and body array coils. Images were acquired using fast T2-weighted HASTE (TE 84 ms/TR 900 ms, coronal, slice 5 mm) sequences at the point of end-expiration and end-inspiration using respiration trigger. Image processing was performed with ImageJ (v1.48, Nat. Institute of Health, USA) and Matlab (Mathworks Inc., Natwick, USA). First, edge detection was performed on all images extracting the diaphragm location. In- and expiration-based images from each slice were merged into a single image (Fig. 3). The displacement of the diaphragm was measured at the marked points in superior–inferior direction.

### Statistical analysis

For all pigs, the end-expiration/end-inspiration difference in diaphragm motion was measured sonographically three times in each experimental step. Mean values and standard deviations were calculated. The results obtained at each experimental step were compared using ANOVA with repeated measurement followed by pairwise comparison procedures. A value of *p* < 0.05 was considered statistically significant.

## Results

**Fig. 4:**
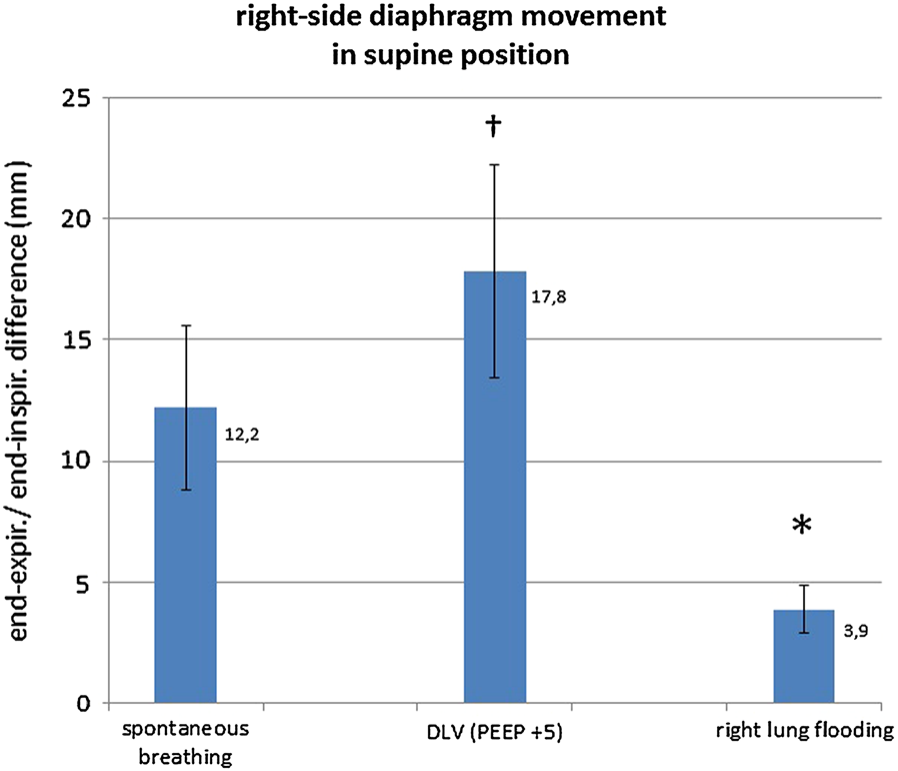
Diaphragm motion of the right hemidiaphragm during spontaneous breathing, during double-lung ventilation (DLV; PEEP +5 cm H_2_O) and after flooding of the right lung wing in supine position. Values depict the mean ± SD of three animals. *Asterisk* indicates that the difference is significant in comparison with DLV (*p* = 0.041). *Dagger* indicates that the difference is significant in comparison with spontaneous breathing (*p* = 0.014)

The end-expiration/end-inspiration difference based on sonographical M-mode measurements of the right hemidiaphragm during spontaneous breathing, mechanical ventilation and after flooding of the right lung is shown in Fig. 4. Double-lung ventilation increased the diaphragm movement in comparison with spontaneous breathing (17.8 ± 4.4 vs. 12.2 ± 3.4 mm, *p* = 0.014). Diaphragm movement on the flooded side during contralateral one-lung ventilation was significantly reduced compared with that during double-lung ventilation (3.9 ± 1.0 vs. 17.8 ± 4.4 mm, *p* = 0.041). Albeit not significant, OLF reduces the movement of the ipsilateral hemidiaphragm in comparison with spontaneous breathing (3.9 ± 1.0 vs. 12.2 ± 3.4 mm, *p* = 0.064).

**Fig. 5:**
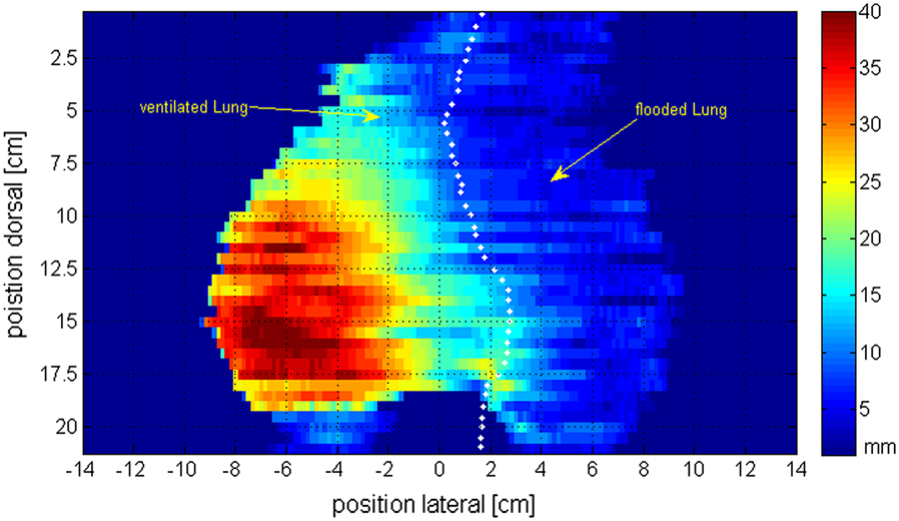
2D presentation of diaphragm displacement between end-expiration and end-inspiration in mm with flooded left lung for a representative single animal. *White dots* show separation between flooded and ventilated lung

**Fig. 6:**
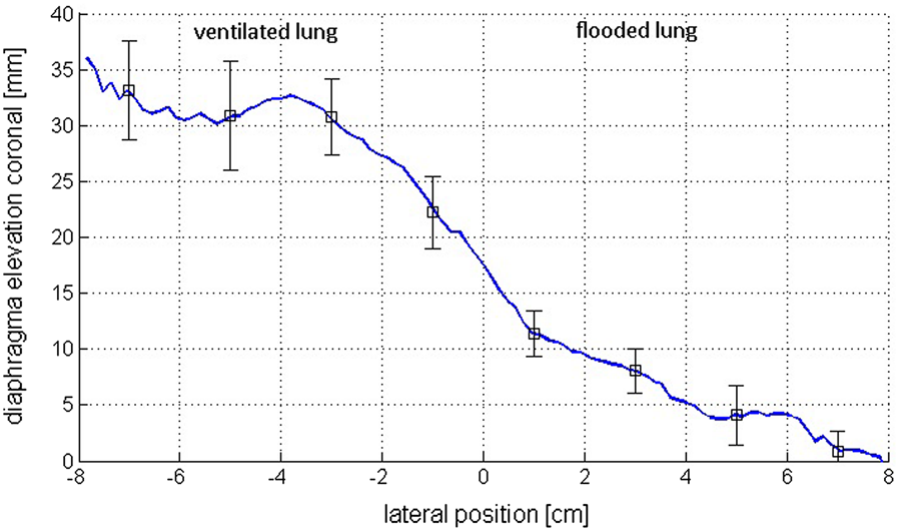
Diaphragmatic displacement between end-expiration and end-inspiration in mm in the coronal plane of the central pulmonary slice (mean ± SD of four animals)

Displacement over the entire diaphragm surface in superior–inferior direction based on MR examination is shown in Fig. 5 for a representative single animal. Further for the diaphragmatic elevation in the central coronal plane for all animals is shown in Fig. 6. The greatest diaphragm movement with up to 35 mm was measured in the laterodorsal diaphragm area on the ventilated side. The movement was continuously reduced toward the flooded side. On the flooded side the greatest diaphragm movement was measured close to the ventilated lung up to 15 mm. The average displacement on the flooded side was 4.2 mm. The lateral parts of the hemidiaphragm on the flooded side showed no movement.

## Discussion

Percutaneous minimal-invasive interventions for local therapy of malignant tumours in lung and upper abdomen is limited by organ motion. As a novel and promising non-invasive intervention, HIFU ablation is being investigated as an option for the treatment of tumours in liver, kidneys and pancreas [15, 16]. HIFU seems also to evolve into a potential new strategy for treating lung cancer [12, 13]. However, organ motion may cause targeting problems and impair the precision and efficacy of the HIFU beam as well as in MR thermometry [17, 18].

Our results show that OLF leads to substantial reduction of the ipsilateral diaphragm motion during mechanical ventilation of the contralateral lung. With ultrasound M-mode we measured a minimal movement (<4 mm) of the hemidiaphragm of the flooded side in comparison with double-lung ventilation and spontaneous breathing (18 and 12 mm, respectively). The MR examinations confirm these results. In contrast to the locally restricted measurement with M-mode sonography, MR imaging allows the measurement of displacement over the entire diaphragm. We found a maximum displacement of 15 mm paramedian close to the ventilated lung and no displacement at the lateral side close to the thoracic wall. The contralateral diaphragm movement due to mechanical one-lung ventilation is partially transferred to the opposite side, while reducing its impact toward lateral sections. The combination of OLF and HFJV of the contralateral lung may provide the opportunity to prevent even slightest movement of the diaphragm and liver.

The effects of OLF for diaphragm motion suppression had never been described before. HFJV and HFOV can reduce the diaphragm motion in a similar extent compared to our results [2, 10]. However, high-frequency ventilation is feasible only in 80 % of patients due to the invisibility of tumours [19]. The air-filled lung covers a significant portion of the upper abdomen, limiting the acoustic window for HIFU application [3].

OLF enables efficient lung sonography and tumour imaging. The procedure is safe in acute and chronical animal experimentations [20, 21]. Furthermore, the flooded lung represents an ideal acoustic path for HIFU insonation into lung tumours [13]. It is plausible that OLF would only be performed if an acoustic access to lung or adjacent organs for sonographic and or therapeutic interventions using HIFU is required. For that, superior conditions arise by the good acoustic pathway and the motion suppression of the ipsilateral hemidiaphragm.

Some limitations are present as well. The pig represents an animal model close to human physiology, but it does not have a mediastinum like in humans. This additional structure is separating the left and right lung wing section. It is therefore likely that in humans the effect of diaphragm motion suppression during lung flooding is more effective than in our presented large animal model. Our M-mode ultrasound measurements were performed on animals placed in supine position. Sonographic imaging of the depending diaphragm is difficult to perform in a lateral decubitus position. MR imaging was performed on animals placed in the lateral decubitus position with flooded lung below, corresponding with the future clinical application. For the MRgHIFU treatment of liver or lung tumours the patients must be placed in the lateral decubitus position because the HIFU transducer is usually integrated in the MR table. In the future, we will investigate the influence of various animal positions on the diaphragm motion and ventilation parameters, including HFJV during OLF.

In conclusion OLF leads to substantial reduction of diaphragm motion on the ipsilateral side with the prospect that targeting and motion compensation algorithms for interventions like HIFU ablation of intrapulmonary and hepatic lesions might not be required.

## Abbreviations

HIFU: high-intensity focused ultrasound
MRgHIFU: magnetic resonance-guided HIFU
HFJV: high-frequency jet ventilation
HFOV: high-frequency oscillatory ventilation
OLF: one-lung flooding
PEEP: positive end-expiratory pressure
FIO_2_: fraction of inspired oxygen
ECG: electrocardiography
TV: tidal volume
SD: standard deviation

## References

[1] Illing RO, Kennedy JE, Wu F, ter Haar GR, Protheroe AS, Friend PJ, Gleeson FV, Cranston DW, Phillips RR, Middleton MR (2005). The safety and feasibility of extracorporeal high-intensity focused ultrasound (HIFU) for the treatment of liver and kidney tumours in a Western population. Br J Cancer.

[2] Fritz P, Kraus HJ, Dölken W, Mühlnickel W, Müller-Nolte F, Hering W (2006). Technical note: gold marker implants and high-frequency jet ventilation for stereotactic, single-dose irradiation of liver tumors. Technol Cancer Res Treat.

[3] Al-Bataineh O, Jenne J, Huber P (2012). Clinical and future applications of high intensity focused ultrasound in cancer. Cancer Treat Rev.

[4] Orsi F, Arnone P, Chen W, Zhang L (2010). High intensity focused ultrasound ablation: a new therapeutic option for solid tumors. J Cancer Res Ther.

[5] Schwenke M, Strehlow J, Haase S, Jenne J, Tanner C, Langø T, Loeve AJ, Karakitsios I, Xiao X, Levy Y, Sat G, Bezzi M, Braunewell S, Guenther M, Melzer A, Preusser T (2015). An integrated model-based software for FUS in moving abdominal organs. Int J Hyperthermia.

[6] Grissom WA, Rieke V, Holbrook AB, Medan Y, Lustig M, Santos J, McConnell MV, Pauly KB (2010). Hybrid referenceless and multibaseline subtraction MR thermometry for monitoring thermal therapies in moving organs. Med Phys.

[7] Wijlemans JW, de Greef M, Schubert G, Bartels LW, Moonen CT, van den Bosch MA, Ries M (2015). A clinically feasible treatment protocol for magnetic resonance-guided high-intensity focused ultrasound ablation in the liver. Invest Radiol.

[8] Celicanin Z, Auboiroux V, Bieri O, Petrusca L, Santini F, Viallon M, Scheffler K, Salomir R (2014). Real-time method for motion-compensated MR thermometry and MRgHIFU treatment in abdominal organs. Magn Reson Med.

[9] Yin F, Kim JG, Haughton C, Brown SL, Ajlouni M, Stronati M, Pamukov N, Kim JH (2001). Extracranial radiosurgery: immobilizing liver motion in dogs using high-frequency jet ventilation and total intravenous anesthesia. Int J Radiat Oncol Biol Phys.

[10] Chaves AH, Cava JR, Simpson P, Hoffman GM, Samyn MM (2013). Infant cardiac magnetic resonance imaging using oscillatory ventilation: safe and effective. Pediatr Cardiol.

[11] Lesser TG, Schubert H, Bischoff S, Wolfram F (2013). Lung flooding enables efficient lung sonography and tumour imaging in human ex vivo and porcine in vivo lung cancer model. Eur J Med Res.

[12] Wolfram F, Boltze C, Schubert H, Bischoff S, Lesser TG (2014). Effect of lung flooding and high-intensity focused ultrasound on lung tumours: an experimental study in an ex vivo human cancer model and simulated in vivo tumours in pigs. Eur J Med Res.

[13] Wolfram F, Reichenbach JR, Lesser TG (2014). An ex vivo human lung model for ultrasound-guided high-intensity focused ultrasound therapy using lung flooding. Ultrasound Med Biol.

[14] Defosse JM. Einfluss von Spontanatmung auf die endexspiratorische Belüftung der Lunge bei tierexperimentellem Lungenschaden. Inaugural-Dissertation. Bonn; 2012.

[15] Wu F, Wang ZB, Zhu H, Chen WZ, Zou JZ, Bai J, Li KQ, Jin CB, Xie FL, Su HB (2005). Feasibility of US-guided high-intensity focused ultrasound treatment in patients with advanced pancreatic cancer: initial experience. Radiology.

[16] Allen M, Rivens I, Visioli A, ter Haar G. Focused ultrasound surgery (FUS): A noninvasive technique for the thermal ablation of liver metastases. In: Ultrasound. PnIST; 2002. p. 17–25.

[17] Jenne JW, Preusser T, Günther M (2012). High-intensity focused ultrasound: principles, therapy guidance, simulations and applications. Z Med Phys.

[18] Salomir R, Viallon M, Kickhefel A, Roland J, Morel DR, Petrusca L, Auboiroux V, Goget T, Terraz S, Becker CD, Gross P (2012). Reference-free PRFS MR-thermometry using near-harmonic 2-D reconstruction of the background phase. IEEE Trans Med Imaging.

[19] Denys A, Lachenal Y, Duran R, Chollet-Rivier M, Bize P (2014). Use of high-frequency jet ventilation for percutaneous tumor ablation. Cardiovasc Intervent Radiol.

[20] Klinzing S, Lesser T, Schubert H, Bartel M, Klein U (2000). One-lung flooding for video-assisted thoracoscopic surgery in animal experiments on pigs—oxygenation and intrapulmonary shunt. Res Exp Med.

[21] Lesser Th, Klinzing S, Schubert H, Kosmehl H (2008). Consequences of one-lung flooding: a histological and immunological investigation. Eur J Med Res.

